# High HIV prevalence and incidence among women in Southern Mozambique: Evidence from the MDP microbicide feasibility study

**DOI:** 10.1371/journal.pone.0173243

**Published:** 2017-03-28

**Authors:** Sibone Mocumbi, Mitzy Gafos, Khatia Munguambe, Ruth Goodall, Sheena McCormack, on behalf of the Microbicides Development Programme

**Affiliations:** 1 Maputo Central Hospital (HCM), Maputo, Mozambique; 2 Manhiça Health Research Centre (CISM), Manhiça District, Mozambique; 3 Medical Research Council Clinical Trials Unit at University College London (MRC CTU at UCL), London, United Kingdom; Leibniz Institute for Prvention Research and Epidemiology BIPS, GERMANY

## Abstract

**Background:**

The study aimed to assess the feasibility of conducting large scale HIV prevention clinical trials in Mozambique by measuring HIV prevalence and incidence among women of reproductive age. This paper describes the baseline socio-demographic characteristics of the Mozambique Microbicides Development Programme (MDP) feasibility cohort, baseline prevalence of HIV and other STIs, and HIV incidence.

**Methods:**

The Mozambique MDP feasibility study was conducted from September 2007 to August 2009 in urban Mavalane and rural Manhiça, in Southern Mozambique. Sexually active, HIV negative women aged 18 years and above were recruited to attend the study clinic every 4 weeks for a total of 40 weeks. At baseline, we collected demographic and sexual behaviour data, samples to test for sexually transmitted infections (STI) and conducted HIV rapid testing. STI and HIV testing were repeated at clinical follow-up visits. We describe HIV prevalence of women at screening, the demographic, behavioural and clinical characteristics of women at enrolment, and HIV incidence during follow-up.

**Results:**

We screened 793 women (369 at Mavalane and 424 at Manhiça) and enrolled 505 eligible women (254 at Mavalane and 251 at Manhiça). Overall HIV prevalence at screening was 17%; 10% at Mavalane and 22% at Manhiça. Women screened at Manhiça were twice as likely as women screened at Mavalane to be HIV positive and HIV positive status was associated with younger age (18–34), lower educational level, not using a reliable method of contraception and being Zionist compared to other Christian religions. At enrolment contraceptive use was low in both clinics at 19% in Mavalane and 21% in Manhiça, as was reported condom use at last sex act at 48% in Mavalane and 25% in Manhiça. At enrolment, 8% of women tested positive for *Trichomonas vaginalis*, 2% for *Neisseria gonorrhoeae*, 4% for *Chlamydia trachomatis* and 46% for *bacterial vaginosis*. In Manhiça, 8% of women had active syphilis at screening. HIV incidence was 4.3 per 100 person years at Mavalane and 9.2 per 100 person years at Manhiça.

**Conclusions:**

We demonstrated the ability to recruit a cohort of women at risk of HIV who were willing to participate in clinical research. The high HIV incidence necessitates additional action around HIV prevention for women and offers opportunities to evaluate the impact of available prevention options, such as treatment as prevention and oral PrEP. The high HIV incidence and STI prevalence also offers opportunities to evaluate the added benefit of potential prevention options such as new formulations of oral PrEP, vaginal microbicides (also called topical PrEP), vaccines, and multi-purpose technologies for HIV, STIs and contraception.

## Introduction

With approximately 1.5 million adults living with HIV in 2015 and an adult HIV prevalence of 10.5%, Mozambique is among the top ten countries with the highest HIV prevalence in the world [1,2]. HIV prevalence is even higher for women of reproductive age at 13%, peaking among women aged 25 to 29 at 17% [3]. Women’s HIV prevalence differs dramatically across the 11 provinces, from 3% in the northern province of Niassa to 30% in the southern province of Gaza [3]. HIV prevalence is higher among urban than rural women at 18% versus 11% [3]. In the areas of interest for this study, Maputo City and Manhiça District, HIV prevalence for women is 21% and 16% respectively [3].

In Mozambique, there is limited data on HIV incidence. Based on UNAIDS estimates there were 100,000 new HIV infections in the country in 2013 [4]. Mozambique still accounted for 8% of all new HIV infections in Sub-Saharan Africa, despite recent reports of a 27% decline in new HIV infections from 2005 to 2013 [1,4]. Low and inconsistent condom use, multiple partners, transactional sex, low rates of male circumcision, and mobility and migration are considered the main drivers of the epidemic in Mozambique [5].

The Mozambican Government has made efforts to address the HIV epidemic and is committed to the UNAIDS Fast-Track Strategy to end its AIDS epidemic by 2030 [6]. However, despite the expansion of HIV testing and treatment services, less than half of all adults in need of antiretroviral therapy received it in 2012 [1]. This proportion drops to less than a quarter of adults when applying the new World Health Organisation 2013 guidelines to initiate treatment when an individuals’ CD4 count falls below 500 cells/μL or immediately for more vulnerable individual’s [1]. Expanded efforts in both HIV prevention and treatment are urgently required to address the HIV epidemic in Mozambique.

Since 2004, the Mozambican Government has supported the principal of new HIV biomedical prevention research and initiatives to establish local research sites for microbicide clinical trials. As a result, a Mozambican partnership between the National Institute for Health (INS), the Manhiça Health Research Centre (CISM) and the Foundation for Community Development (FDC) joined the Microbicides Development Programme (MDP) [7,8]. This partnership hosted the Mozambique MDP feasibility study in the Mavalane area of Maputo City and Manhiça District, in southern Mozambique. The study aimed to assess the feasibility of conducting large scale biomedical HIV prevention clinical trials in Mozambique by measuring HIV prevalence and incidence. This paper describes the baseline socio-demographic and behavioural characteristics of the Mozambique MDP feasibility cohort, baseline prevalence of HIV and other sexually transmitted infections (STIs), and HIV incidence.

## Methods

The Mozambique MDP feasibility study was conducted from September 2007 to August 2009. The study recruited at two dedicated research clinics located within the grounds of National Health Service (NHS) facilities, at 1 de Junho Health Centre in the peri-urban Mavalane area of Maputo City and at Manhiça Health Centre in the rural Manhiça District which is approximately 80km north of Maputo City. The study aimed to recruit approximately 500 women across both centres with 40 weeks of follow-up. The sample size was based on the precision of the estimate of HIV incidence per 100 women years; a 5% incidence of HIV and 350 women years at risk (approx. 500 women) gives a one-sided confidence interval width of 2.39.

Information about the study was disseminated at community mobilisation meetings, via local stakeholders, and at the NHS clinic facilities. Women were invited to attend the research clinics to find out more about the study and to be screened for eligibility. At the initial visit, women who provided informed consent were screened against the following eligibility criteria: 18 years of age or above, HIV negative, not pregnant, sexually active in the last month, willing and able to give informed consent, and willing to undergo regular urine pregnancy tests, HIV testing, and speculum examinations. Demographic and baseline behaviour data were collected, HIV counselling was provided, HIV rapid tests were performed, and blood samples were collected for syphilis testing. Data on the use of any family planning method were collected and for the purposes of this analysis we defined ‘reliable contraceptives’ as the oral contraceptive pill, injectable Depot medroxyprogesterone acetate (DMPA), the intrauterine contraceptive device (IUCD), and female sterilisation. Eligible women were invited to attend an enrolment visit within 6 weeks.

At enrolment, a genital examination was conducted during which endocervical samples were collected for *Neisseria gonorrhoeae* (NG) and *Chlamydia trachomatis* (CT), and a high vaginal swab for *Trichomonas vaginalis* (TV) and Bacterial vaginosis (BV). Women with symptoms and clinical signs of STIs were managed according to Mozambican syndromic management guidelines and all diagnosed STIs were treated [9]. Women who tested HIV positive at screening or during follow up were referred to clinical care which included the provision of anti-retroviral therapy.

Participants were asked to attend the study clinic every 4 weeks for a total of 40 weeks. Participants who missed a scheduled follow-up visit were contacted either by phone or at the location address they provided, up to three times by the tracking team. HIV rapid tests and genital examinations were conducted at clinic visits 12, 24 and 40 weeks after enrolment, with STI tests repeated at week 24. HIV status was assessed using the MDP parallel dual rapid test algorithm performed by study nurses (Abbott Determine HIV1/ 2 test and Trinity Biotech Uni-Gold). The MDP contracted reference laboratory in South Africa (Contract Laboratory Services (CLS), the School of Pathology, University of the Witwatersrand, Johannesburg) confirmed all HIV sero-converters using the standard MDP algorithm which involved two different Enzyme Immunoassays (EIA) and a confirmatory positive HIV qualitative DNA PCR, P24 EIA or HIV-1 Western Blot [10]. Syphilis serology was done by rapid plasma reagin (RPR) (Omega Diagnostics) and TPHA; active syphilis was defined by positivity in both tests. NG and CT was evaluated through polymerase chain reaction (PCR), and TV was evaluated using In-Pouch [11]. BV samples were collected using the Dacron swab, the slides were Gram stained and BV was diagnosed using the Ison Hay method [12]. HIV and STI samples were placed in the respective transport medium and transported to the respective laboratories under temperature-controlled conditions, and from there onwards to CLS in batches as required. Syphilis and TV tests were conducted at the laboratory of the 1 de Junho Health Centre and the CISM laboratory for Mavalane and Manhiça respectively, and NG and CT tests were conducted at CLS. At enrolment, clinic staff failed to collect samples for TV, NG/CT and BV from 10%, 7%, and 2% of participants, and this increased at the week 24 visit to 22%, 15% and 16% who attended the visit, respectively.

Data were double entered into a common SQL database at each site. Demographic and behavioural characteristics were tabulated to consider differences by HIV status at screening. Differences between HIV negative and positive women were examined by calculating chi-square tests and fitting logistic regression models. Differences that attained statistical significance at the 10% level using a likelihood ratio test (LRT) were considered for the multivariate model. We considered differences to be statistically significant based on a 95% confidence interval. Demographic, behavioural and clinical characteristics of women at enrolment were tabulated. T-tests were used to compare means. STI point prevalence at weeks 0 and 24 (number with positive result/total number tested) and HIV incidence (number of new HIV+ diagnoses/women years at risk) were calculated. We estimated the time of HIV seroconversion as the midpoint between the last negative and first positive HIV tests. Data were analyzed using Stata 14 (StataCorp, College Station, Texas, USA).

Approval was obtained from the Mozambique National Ethics Committee (CNBS) (approval number 16/CNBS/07). Written informed consent, supported by a comprehension test, was obtained from all participants. Participants were not given a remittance for the study visits, but were offered transport in Manhiça or reimbursed the cost of public transport in Mavalane, and offered food and drink during the study visits at both sites.

## Results

### Women screened

**Fig 1:**
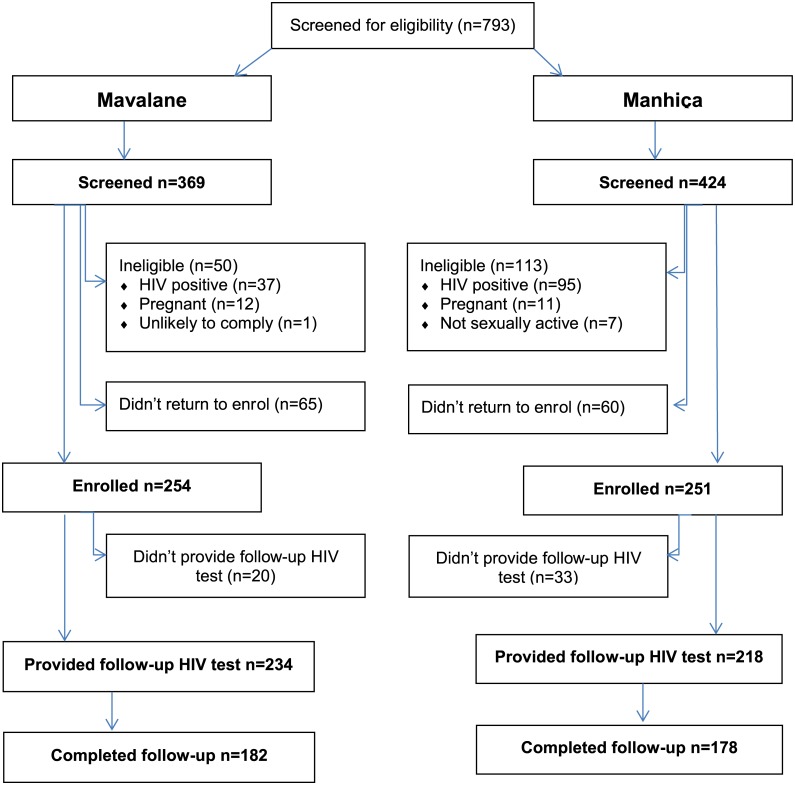
Consort diagram.

Seven-hundred and ninety-three (793) women attended a screening visit, 369 (47%) at Mavalane and 424 (53%) at Manhiça. One-hundred and sixty-three (163) women (21%) were ineligible, 132 (17%) who were HIV positive, 23 (3%) who had a positive pregnancy test at screening or enrolment, and 8 (1%) who were not sexually active or unlikely to comply with the protocol (Fig 1). Another 125 (16%) were identified as eligible but did not return for enrolment. A total of 505 eligible women enrolled in the study giving a screening to enrolment ratio of 1.57.

### HIV prevalence of women at screening and associated socio-demographic characteristics

HIV prevalence at screening differed substantially between the sites, at 10% (37/369) at Mavalane and 22% (95/424) at Manhiça. As shown in Table 1, a higher proportion of HIV positive women were aged between 18 to 34 years old, had not completed primary education, were Zionist, not using reliable contraception and had not used a condom at last sex. These variables were all associated with HIV status at screening. The majority of women were unemployed and this did not differ by HIV status. Similarly the majority of women reported being in long-term stable relationships (97%) and this did not differ by HIV status (p-value 0.790).

**Table 1:** HIV status at screening by selected demographic and sexual behavioural characteristics.

	Mozambique cohort			p-value	Mavalane—Urban		Manhiça—Rural	
	All	HIV-	HIV +		HIV -	HIV +	HIV -	HIV +
N screened, n (%)	793 (100)	661 (83)	132 (17)		332 (90)	37 (10)	329 (78)	95 (22)
Clinic								
•Mavalane	369 (47)	332 (50)	37 (28)	<0.001				
•Manhiça	424 (53)	329 (50)	95 (72)					
Age								
•18–24^1^	358 (46)	311 (47)	47 (36)	0.010	224 (68)	20 (56)	87 (27)	27 (29)
•25–34	220 (28)	168 (26)	52 (40)		56 (17)	11 (31)	112 (35)	41 (44)
•35–44	124 (16)	106 (16)	18 (14)		26 (8)	2 (6)	80 (25)	16 (17)
•45+	83 (10)	70 (11)	13 (10)		25 (8)	3 (8)	45 (14)	10 (11)
Missing	8	6	2		1	1	5	1
Mean age (SD) ttest	29.0 (10.1)	29.0 (10.3)	29.4 (9.0)	0.657	25.4 (8.9)	27.0 (8.6)	32.6 (10.4)	30.3 (9.0)
Education								
•Did not complete primary	329 (42)	254 (38)	75 (57)	<0.001	68 (20)	18 (49)	186 (57)	57 (60)
•Completed primary or further	462 (58)	406 (62)	56 (43)		264 (80)	19 (51)	142 (43)	37 (40)
Missing	2	1	1		0	0	1	1
Employment status								
•Employed (full or part time)	162 (20)	141 (21)	21 (16)	0.167	52 (16)	7 (19)	89 (27)	14 (15)
•Unemployed	629 (80)	519 (79)	110 (84)		280 (84)	30 (81)	239 (73)	80 (85)
Missing	2	1	1		0	0	1	1
Religion								
•Christian	636 (80)	540 (82)	96 (73)	<0.001	284 (86)	29 (79)	256 (79)	67 (71)
•Zionist	101 (13)	71 (11)	30 (23)		23 (7)	6 (16)	48 (15)	24 (26)
•Other	51 (7)	46 (7)	5 (4)		25 (8)	2 (5)	21 (6)	3 (3)
Missing	5	4	1		0	0	4	1
Reliable contraceptive								
•Any^2^	181 (23)	162 (25)	19 (14)	0.011	89 (27)	9 (24)	73 (22)	10 (11)
•None	612 (77)	499 (75)	113 (86)		243 (73)	28 (76)	256 (78)	85 (89)
Condom use at last sex act								
•No	530 (67)	428 (65)	102 (77)	0.005	180 (54)	29 (78)	248 (75)	73 (77)
•Yes	263 (33)	233 (35)	30 (23)		152 (46)	8 (22)	81 (25)	22 (23)

^1^ Age was calculated from the screening visit at which point one woman was 17 years of age, but was enrolled one day after her 18^th^ birthday.

^2^ Of 181 women, 117 reported oral hormonal contraceptive, 54 reported DMPA, 10 reported other reliable methods which were tubal ligation and the intrauterine contraceptive device.

The numbers of HIV positive women within each site allowed only descriptive statistics (Table 1). However it is noteworthy that women screened at Manhiça were older than women screened at Mavalane with a mean age of 32.1 compared to 25.6 (ttest, p-value<0.001) and that a higher proportion of women in Manhiça were Zionist. In addition, a higher proportion of HIV negative women in Mavalane had higher levels of education and reported condom use at last sex than HIV negative women in Manhiça. A higher proportion of HIV positive women in Manhiça were not using reliable contraception compared to positive women in Mavalane.

In a multivariate model, women screened at Manhiça were twice as likely as women screened at Mavalane to be HIV positive when controlling for age, education, religion and contraceptive use (Table 2). Women aged 25 to 34 compared to 18 to 24 year olds, women who had not completed primary education compared to those who had, women who reported being Zionist compared to other Christian belief systems, and women who reported not using a reliable contraceptive method compared to those who did, were more likely to be HIV-positive (Table 2). Condom use at last sex act was not significantly associated with HIV status in the multivariate models (LRTest p = 0.096).

**Table 2:** Multivariate model assessing characteristics of HIV positive women at screening.

	HIV-	HIV +	LRT p-value	AOR (95% CI)	p-value
N screened, n (%)	661 (83)	132 (17)			
Clinic					
•Mavalane	332 (50)	37 (28)	<0.001	1.00	0.002
•Manhiça	329 (50)	95 (72)		2.05 (1.29, 3.27)	
Age					
•18–24	311 (47)	47 (36)	<0.001	1.00	0.004
•25–34	168 (26)	52 (40)		1.35 (0.82, 2.22)	
•35–44	106 (16)	18 (14)		0.49 (0.25, 0.96)	
•≥45	70 (11	13 (10)		0.51 (0.24, 1.07)	
Education					
•Completed primary of further	406 (62)	56 (43)	0.001	1.00	0.004
•Did not complete primary	254 (38)	75 (57)		2.00 (1.25, 3.18)	
Religion					
•Christian	540 (82)	96 (73)	0.034	1.00	0.036
•Zionist	71 (11)	30 (23)		1.83 (1.09, 3.08)	
•Other	46 (7)	5 (4)		0.62 (0.23, 1.62)	
Reliable contraceptive					
•Any	162 (25)	19 (14)	0.001	1.00	0.003
•None	499 (75)	113 (86)		2.33 (1.33, 4.17)	

### Socio-demographic and sexual behaviour characteristics of women enrolled

Of the 793 women screened, 505 (64%) were eligible and consented to enrol in the study; 254 (50%) at Mavalane and 251 (50%) at Manhiça (Table 3). The mean age of women recruited into the cohort was 29.3 years (SD 10.2 years; range 17–59). Women in Mavalane were on average 6.6 years younger than women in Manhiça. A higher proportion of women enrolled in Mavalane than in Manhiça completed primary education or were unemployed. More women in Manhiça reported their religion as Zionist, while more women in Mavalane reported belonging to other Christian belief systems, compared to the other site. The dominant language was Portuguese in Mavalane and Changana in Manhiça (the main local dialect of Southern Mozambique). The majority of women in Mavalane lived in a household headed by a parent, with a higher proportion of women enrolled in Manhiça than in Mavalane living in households headed by their partner or themselves. Most women lived in households with two people sleeping per room, although a higher proportion of women in Manhiça lived in households were four people slept per room compared to Mavalane.

**Table 3:** Demographic characteristics at enrolment.

	Mavalane	Manhiça	p-value
N enrolled, n (%)	254 (50)	251 (50)	
Age			<0.001
•18–24	169 (67)	58 (23)	
•25–34	37 (15)	91 (37)	
•35–44	23 (9)	67 (27)	
•45+	24 (9)	31 (13)	
Missing	1	4	
Mean (SD) ttest	26.0 (9.57)	32.6 (9.70)	<0.001
Education			<0.001
•Did not complete primary	51 (20)	145 (58)	
•Completed primary of further	203 (80)	106 (42)	
Employment status			<0.001
•Employed (full/part-time)	36 (14)	69 (27)	
•Unemployed	218 (86)	182 (73)	
Religion			0.041
•Christian	219 (86)	9)	
•Zionist	19 (8)	36 (15)	
•Other	16 (6)	15 (6)	
Missing		3	
Language			<0.001
•Changana	93 (37)	1)	
•Portuguese	146 (58)	39 (16)	
•Other	14 (5)	7 (3)	
Missing	1	3	
Head of household			<0.001
•Partner	75 (30)	5)	
•Self	10 (4)	47 (19)	
•Parent or other family	169 (67)	65 (26)	
Number of people per room			<0.001
•4 or more people per room	31 (12)	59 (24)	
•3 people per room	72 (28)	68 (27)	
•2 people per room	140 (55)	101 (40)	
•1 person per room	11 (4)	23 (9)	
Reliable contraceptive			0.533
•Any*	48 (19)	53 (21)	
•None	206 (81)	198 (79)	

*23 Depot medroxyprogesterone acetate (DMPA), 72 oral pills, 5 Intrauterine Device and 1 female sterilization.

The use of reliable contraceptives at enrolment was low in both clinics at 19% in Mavalane and 21% in Manhiça, with no significant difference between the clinics (Table 3). The main methods of contraception were the oral pill (Mavalane 15%, Manhica 13%) and the depot medroxyprogesterone acetate (DMPA) injectable (Mavalane 2%, Manhiça 7%). Women reported low levels of sexual activity in the week before enrolment (median 1, IQR 0–1, range 0–5) and only 6 women reported having a casual rather than a long-term partner. Forty-eight percent of women enrolled in Mavalane and 25% in Manhiça reported using a condom at their last sex act, and the difference between the clinics reached statistical significance (p>0.001). Eighty-eight percent of women (443) reported being aged 15 to 19 years old the first time they had sex, with only 5% (25) younger and 7% (35) older (no data for 2 women).

At enrolment, 8% of women tested positive for TV, 2% for NG, 4% for CT and 46% for BV (Table 4). There were no statistically significant differences between the sites. In Manhiça, 8% (21/250) of women had active syphilis, although results are not available at Mavalane.

**Table 4:** STIs at baseline.

N, (%)	Total	Mavalane	Manhiça
TV	37/456 (8)	13/213 (6)	24/243 (10)
NG	10/470 (2)	7/227 (3)	3/243 (1)
CT	18/470 (4)	7/227 (3)	11/243 (5)
BV (I&H 3 = positive)	226/494 (46)	117/249 (47)	109/245 (44)
Syphilis^1^		----	21/250 (8)

^1^Result from screening visit

Women in Mavalane lived in households with indicators of higher socio-economic status than women in Manhiça (Table 5), as indicated by a higher proportion of Mavalane households having electricity, running water, a radio, television, refrigerator, computer, bicycle, car and even mosquito net.

**Table 5:** Socio-economic characteristics at enrolment: Proportion of women who reported household ownership of assets.

	Mavalane	Manhiça	p-value
N enrolled, n (%)	254 (50)	251 (50)	
Household electricity	225 (89)	105 (42)	<0.001
Household water tap	166 (65)	74 (29)	<0.001
Radio	214 (84)	137 (55)	<0.001
Television	225 (89)	98 (39)	<0.001
Refrigerator	178 (70)	78 (31)	<0.001
Computer	46 (18)	9 (4)	<0.001
Bicycle	37 (15)	56 (22)	0.025
Car	59 (23)	35 (14)	0.007
Mosquito net	162 (64)	129 (51)	0.005

### Follow up data on sexual behaviour, STI prevalence and HIV incidence

In total, 360 (71%) women completed follow-up (≥38 weeks) and as shown in Fig 1 this was similar in both clinics (72% in Mavalane and 71% in Manhiça). The median weeks of follow up were week 40 (interquartile range IQR 24, 40) in Mavalane, and week 40 (IQR 32, 40) in Manhiça. However, only 50% of women in Mavalane (127/254) and 56% (141/251) of women in Manhiça attended 75% (3/4) of the scheduled clinical follow-up visits.

During follow-up, few women reported concurrent multiple partners (n = 4), having sex during menstruation (n = 11), intravaginal cleansing or insertion (n = 24), or anal sex (n = 15).

Although 324 women (173 in Manhiça and 151 in Mavalane), attended the week 24 visit, STI test results were not available for almost 20% of them. Based on this limited data, TV prevalence was 7% (18/253), NG prevalence was 3% (7/274) and CT prevalence was 1% (4/270).

Of the 505 women enrolled, follow-up HIV tests were available for 452 (90%) women (234 in Mavalane and 251 in Manhiça). There were 21 new HIV infections in 315 person years of follow up with an overall incidence of 6.66 per 100 person years (95% CI: 4.34, 10.22). There were 7 infections at Mavalane in 163 person years of follow up with a HIV incidence of 4.29 per 100 person years (95% CI: (2.04, 8.99) and 14 at Manhiça in 152 person years of follow up with a HIV incidence of 9.21 per 100 person years (95% CI: 5.46, 15.55).

## Discussion

In this paper we have presented the baseline characteristics of the Mozambique MDP feasibility study, which was the first such study in Mozambique. We demonstrated the ability to recruit a population of women at risk of HIV who were willing to participate in clinical research. A subsequent study was conducted in the Sofala province of northern Mozambique which showed high levels of willingness to participate in HIV prevention trials among women [13].

In this study the screening to enrolment ratio was favourable when compared to the other MDP feasibility studies (with 33% ineligibility) and the MDP301 phase III clinical trial (with 30% ineligibility) conducted in South Africa, Tanzania, Uganda and Zambia [7,8,14]. The proportion of eligible women who did not return for enrolment was similar to the other MDP feasibility studies (approximately 18%), but higher than in the MDP 301 clinical trial when women were offered a vaginal product to use (7%) [7]. It is encouraging that the rate of eligibility in Mozambique was not lower than in the other feasibility studies [7]. In Mozambique, eligible women who chose not to return for enrolment were not tracked as this was considered an active refusal to consent. It is not surprising that the return rates were similar across the other MDP feasibility studies which were also not interventional, yet lower compared to interventional clinical trials where the motivation to participate is likely to be higher.

Rates of study ineligible are mainly driven by HIV prevalence at screening. Baseline HIV prevalence in Mavalane was lower than at other MDP centres. However HIV prevalence in Manhiça was comparable to the HIV prevalence reported in the MDP feasibility studies and clinical trial in Johannesburg in South Africa (19–20%) and Mwanza in Tanzania (20–25%), but lower than in Mazabuka in Zambia (29–30%), Durban in South Africa (36–47%) and the Africa Centre in South Africa (28–50%) [7,8,14].

In Mavalane, the HIV prevalence observed among women in the MDP feasibility study (10%) was lower than expected based on the national surveillance estimate of 21% in Maputo City [3]. In Manhiça, although the HIV prevalence (22%) was higher than the provincial average (16% in 2009), it was similar to that reported in the same district in HIV testing centres (23% in 2004–5) and ante-natal clinics (29% in 2010), although lower than among women in a cross-sectional survey (43% in 2012) [3,15,16]. The lower than anticipated HIV prevalence at screening may indicate that the recruitment messages were successful in terms of only attracting women who thought they were HIV negative, although we did not collect data on HIV testing history.

As expected, HIV prevalence peaked among women below 34 years old, consistent with other studies [3,17,18]. We found an association between lower education and higher HIV prevalence as seen in some studies [17,18] but not observed in the national surveillance programme [3]. We found that women who reported using a reliable contraceptive method were less likely to be HIV positive at screening than women not using a reliable method, which is similar to other data in Mozambique which has either observed no difference or an association in the same direction [17,18]. In our study we saw a higher risk of HIV prevalence among women who reported being Zionist although there is no data to compare this with in Mozambique.

Condom use at last sex act was low, especially in Manhiça. However, the national survey reported only 8% of women used a condom at last sex act in 2009 although the rate was higher at 44% among unmarried women [3]. Few of the other cohort studies in Mozambique have reported on condom use at last sex, but have reported condom use as the main contraceptive method used by between 8% to 21% of women [13,17,18]. The highest reported use has been among 15 to 24 year old women in a national survey conducted in 2001 (71%) [19]. It is noteworthy that in our study, reported condom use at last sex act was not associated with HIV infection which is indicative of the limitations of using condom use at last sex as a measure of consistent use.

Longitudinal HIV incidence data is limited in Mozambique with approximately five reported studies across the country [13,17,20–22]. Incidence data among women has been reported in post-partum clinics at 2.9 per 100 person-years in Maputo Province and at 3.6 in Gaza Province, at 4.6 in a cohort study in Gaza, at 4.9 from cross-sectional surveys in Manhiça, at 6.5 in a cohort study in Sofala, and as high as 14.1 based on consecutive prevalence surveys in Manhiça [13,17,20–22]. Participants were similar across the studies, as sexually active women of reproductive age, although with the exception of the latter referenced study in Manhiça, women in the other studies tended to be slightly younger than in our study with mean ages below the mid-20s, more comparable to the Mavalane than the Manhiça cohort. The overall HIV incidence reported in this study (6.7 per 100 person years) was at the upper end of incidence estimates, and the incidence reported in Manhiça at 9.2 per 100 person years is consistent with the higher rates in this province which are thought to be stabilising [22].

The weighted estimate of HIV incidence in the MDP feasibility studies in South Africa, Tanzania, Uganda and Zambia was 6.2 per 100 woman years ranging from 3.5 to 12.6 [7]. The overall HIV incidence in the MDP 301 clinical trial was 4.6 per 100 woman years ranging from 1.5 to 6.1 [8]. The HIV incidence observed in Mozambique demonstrates that both Mavalane and Manhiça are feasible sites for the conduct of large scale HIV prevention clinical trials.

There is also limited data on STI prevalence in Mozambique. In this study, NG was lower, CT was higher, and TV and syphilis was similar to that reported in the other MDP 301 trial sites among HIV negative women (NG 3%, CT 8%, TV 9%, syphilis 4%) [8]. Our findings are also comparable to those reported in a 2004 survey in the Tete province for NG (2.5%) and CT (4.1%) although higher for syphilis (5%) [23]. However, the prevalence of STIs in our study are substantially lower than in a 2000 survey among women enrolled predominantly at ante-natal clinics, as well as family planning clinics and in the community in Manhiça (NG 14%, CT 8%, TV 31%, syphilis 12%) [24]. The reasons for the very high prevalence observed in the 2000 survey are not clear, although 12% of the sample was HIV positive and there was a non-significant trend for the presence of all STIs in HIV positive women.

This was the first study to evaluate the feasibility of conducting a clinical trial of vaginal microbicides in Mozambique. The findings demonstrate that both centres were able to recruit and follow-up women with high HIV incidence in a non-interventional cohort study. In Mavalane, the HIV prevalence was lower than at the other MDP research centres while the HIV incidence was comparable. Whereas in Manhiça, the HIV prevalence was similar to the other MDP research centres while the HIV incidence was much higher. These criteria make conducting large-scale biomedical HIV prevention trials in these communities highly feasible.

The main limitation of this study was that the retention rates were not sufficient for a phase III clinical trial, although we expect retention could be improved with the offer of an investigational product as we observed in the other MDP centres. Another limitation of the study was the sub-optimal collection of STI samples as per the protocol schedule. Our ability to measure STI prevalence during follow-up was hindered by both poor retention and inefficient follow up for specimen collection, both of which could be improved in future studies. A further limitation was that we did not collect marital status, involvement in transactional or commercial sex, parity, or alcohol or substance use. Marital status and involvement in transactional sex have been shown to be associated with HIV infection in Mozambique [3,18].

Both HIV prevalence and incidence remain unacceptably high in these two areas of Mozambique but especially in the Manhiça area which is located on the transport route from Maputo to Beira. There is an urgent need to expand both ART treatment and prevention options for people in Mozambique, including the provision of oral pre-exposure prophylaxis [25]. In parallel there is continued need for community mobilisation around HIV testing, prevention and treatment, STI prevention and treatment, and reliable family planning options. In the meantime we need additional data on HIV and STI prevalence and incidence in other provinces of Mozambique and continued monitoring nationally to understand the epidemics and the potential impact of any new prevention modalities. Our findings support the feasibility of conducting HIV prevention trials in Mozambique and enrolling a cohort of women at particularly high risk of HIV acquisition. The high HIV incidence necessitates additional action around HIV prevention and offers opportunities to evaluate the impact of available prevention options such as treatment as prevention and oral PrEP. The high HIV incidence and STI prevalence also offers opportunities to evaluate the added benefit of potential prevention options such as new formulations of oral PrEP, vaginal microbicides (also called topical PrEP), HIV vaccines, and multi-purpose technologies for HIV, STIs and contraception.
